# The effect of daily intake of vitamin D-fortified yogurt drink, with and without added calcium, on serum adiponectin and sirtuins 1 and 6 in adult subjects with type 2 diabetes

**DOI:** 10.1038/s41387-021-00168-x

**Published:** 2021-07-30

**Authors:** Bahareh Nikooyeh, Bruce W. Hollis, Tirang R. Neyestani

**Affiliations:** 1 Laboratory of Nutrition Research, National Nutrition and Food Technology Research Institute and Faculty of Nutrition Sciences and Food Technology, Shahid Beheshti University of Medical Sciences, Tehran, Iran; 2 Division of Neonatology, Department of Pediatrics, Medical University of South Carolina, Charleston, SC USA

**Keywords:** Type 2 diabetes, Hormones

## Abstract

**Background:**

Some evidence suggests indirect ameliorating effects of vitamin D in diabetes via adiponectin and sirtuins. This study aimed to evaluate the effects of daily intake of vitamin D-fortified yogurt drink, either with or without added calcium, on serum adiponectin, sirtuins (SIRT)1 and 6.

**Methods:**

Briefly, 75 adults aged 30–60 years from both sexes with type 2 diabetes were randomly allocated to one of the three groups: (i) D-fortified-yogurt drink (DY; containing 1000 IU vitamin D and 300 mg calcium), (ii) Ca+D-fortified-yogurt drink (CDY; containing 1000 IU vitamin D and 500 mg calcium) and (iii) plain yogurt drink (PY; containing no detectable vitamin D and 300 mg calcium). All assessments were performed initially and after 12 weeks.

**Results:**

A significant within-group increment in serum adiponectin concentrations was observed in both DY and CDY groups (+60.4 ± 8.6, +57.5 ± 6.4 µg/L, respectively; *p* < 0.001 for both). The concentrations of SIRT1 and SIRT6 had a significant within-group increment only in the CDY group (*p* = 0.003, *p* = 0.001 respectively). Being in CDY group was more favorable predictor of improvement in SIRT6 concentrations. Changes of 25(OH)D were a significant predictor of changes of adiponectin. However, this association disappeared following adjustment for changes of SIRT1. In contrast, the association between changes of 25(OH)D and HbA1c remained significant even after adjustment for SIRT1.

**Conclusions:**

Daily consumption of vitamin D-fortified yogurt drink for 12 weeks resulted in an increase in circulating concentrations of SIRT1 and SIRT6 in T2D subjects and D+Ca-fortified yogurt drink was more in favor of SIRT6 increment.

## Introduction

Diabetes is the most prevalent metabolic disease globally. The number of people with diabetes is estimated to increase from 463.0 million in 2019 to 700.2 million in 2045 with faster rate in low- and middle-income countries than in high-income countries [1]. This disease is accompanied by several devastating complications including cardiovascular disease (CVD), nephropathy, neuropathy, depressed immunity, impotence and infertility, stroke, retinopathy, cataracts, myocardial infarction and premature death [2]. Over 90% of diabetes cases are type 2 (T2D) which is predisposed by positive family history, central obesity, and sedentary lifestyle. Therefore, T2D is partly preventable or at least can be remarkably delayed by weight control, healthy diet, and increased physical activity [3]. The importance of prevention of diabetes and its complications lies in the health care, social as well as economic burden of this disease [4–6].

While weight control and healthy lifestyle including healthy diet still consist the core of T2D treatment [7], attempts have been made to find an alternative therapy, including dietary and micronutrient supplements, to control blood glucose in the subjects with diabetes [8, 9]. Among the micronutrients, vitamin D has attracted more attention as it may be associated with both onset and treatment of T2D and consequently it has been, and continues to be, the subject of countless studies [10–15]. The other facet is the global high prevalence of vitamin D deficiency, its relation with health [16] and the necessity of reaching to vitamin D adequacy through appropriate interventions including supplementation and food fortification [17].

With the discovery of vitamin D-binding receptors in various cells and tissues, interest has increased concerning the potential health effects of vitamin D deficiency on the proper functioning of many systems in the body including the immunity, cardiovascular system, vision, the growth and division of cells and the formation of blood vessels. Several reports have confirmed the connection of hypovitaminosis D and the occurrence of many diseases including rheumatoid arthritis, metabolic disorders, cancer, depression, and T2D [18–20]. The ameliorating effects of raising vitamin D status of subjects with T2D on glycemic status as well as parathyroid hormone, some antioxidative and inflammatory biomarkers have been already documented [11, 14, 15, 21–23]. Though the regulating effect of vitamin D on pancreatic β-cell function and insulin signaling has been described [24], there is evidence suggesting an indirect effect of vitamin D in T2D through other mediators including adiponectin and sirtuins [14, 25].

Adiponectin is an adipokine secreted mainly by adipose tissue and also by muscle [26] involving in the regulation of blood glucose and lipids [27]. Adiponectin can improve insulin resistance via several mechanisms including suppressing pro-inflammatory cytokines and oxidative stress, reducing hepatic glucose production and increasing glucose uptake and utilization by skeletal muscle cells [27].

Sirtuins, a family of highly conserved nicotinamide adenine dinucleotide (NAD^+^)-dependent enzymes that modify histones and some other proteins post-translationally, are related to aging and longevity [28]. However, a growing body of evidence indicates a role for sirtuins in insulin resistance, inflammation, and oxidative stress in diabetes [29–34]. Even sirtuins have been proposed as a target in the treatment of T2D [35]. Among seven mammalian sirtuins, sirtuin 1(SIRT1) and 6 (SIRT6) have been reported to have more relevance to glucose homeostasis in T2D [36, 37]. It has been shown that SIRT6 deficiency may impair glucose tolerance and pancreatic β-cell function [38]. SIRT1, on the other hand, may increase insulin sensitivity through inhibiting protein tyrosine phosphatase 1b which has an inhibitory effect on the insulin receptors [39]. This study was, therefore, undertaken to evaluate (i) if regular daily intake of vitamin D-fortified yogurt drink affects serum concentrations of adiponectin, SIRT1 and SIRT6 and (ii) if consuming added calcium to D-fortified yogurt drink influences these effects.

## Methods

### Subjects and study design

We used serum samples kept at our biobank and data from a previously reported clinical trial registered at clinicaltrials.gov as NCT01229891 [15]. The sample size was calculated using the software G power 3.19.2 [40], based on 80% of power and the effect size of 0.4. It was determined that a minimum of 66 participants was required. Using random numbers generator in Excel software (version 2007), the main researcher randomly selected seventy-five participants (30 men and 45 women) from our databank. As described earlier [15], our subjects were initially recruited from the registered T2D patients at the Iranian Diabetes society. Eligible study participants were adults with confirmed T2D aged 30–60 years. Pregnant or lactating women as well as subjects with regular intake of nutritional supplements during the three months prior to the study and those with any clinical diseases affecting vitamin D metabolism were excluded.

The interventions consisted of a 12 week, randomized, placebo-controlled, double-blind trial conducted during late fall and winter when dermal vitamin D synthesis is minimal [41]. The participants were randomly assigned to one of the three groups: (i) D-yogurt drink (DY), consumed as part of their usual diet 500 mL/day D-fortified yogurt drink containing 1000 IU vitamin D and 300 mg naturally occurring calcium (*n* = 25), (ii) Ca+D-yogurt drink (CDY), consumed 500 mL/day fortified yogurt drink containing 1000 IU vitamin D and 500 mg calcium (*n* = 25) and (iii) plain (unfortified) yogurt drink (PY) consumed 500 mL/day unfortified yogurt drink containing no detectable vitamin D and 300 mg calcium (*n* = 25). Yogurt drinks were in 250 mL bottles so participants had to drink two bottles a day, preferably one with lunch and one with dinner. From the beginning to the end of the interventions and analyses, only the main investigator was aware of the group allocations and both participants and other members of the research team were blinded.

All procedures were approved by the Research Ethics Committee of National Nutrition and Food Technology Research Institute (NNFTRI). All subjects signed an informed written consent.

### Assessments

All measurements including dietary, anthropometric, and laboratory assessments performed before and after intervention were described in detail elsewhere [14, 15]. In the original project, dietary intake was evaluated using 24-h recall questionnaire for two days (including a weekend). Weight and height were measured using standard methods to the nearest of 0.1 kg and 0.1 cm, respectively. Body mass index was calculated by dividing weight (kg) by height^2^ (m).

Serum samples were obtained from our biobank. Serum concentrations of 25-hydroxycalciferol (25(OH)D) were measured using high-performance liquid chromatography (HPLC) [42] at the Laboratory of Nutrition Research that has been participating in the Vitamin D External Quality Assessment Scheme (DEQAS, www.DEQAS.org.uk) since 2012. Methods of analysis for glycated hemoglobin (Hb A1c), adiponectin, and percent of total body fat mass (FM) have been described elsewhere [14, 15].

We measured circulating concentrations of SIRT1 and SIRT6 using enzyme immunoassay (EIA) method and commercial kits according to the manufacturer protocols (both from ZellBio, Veltlinerweg, Ulm, Germany) and a microplate reader (Statfax 3200; Awareness Technology, Inc., Palm City, FL).

### Statistical analyses

All data are presented as means ± standard deviation (SD) or 95% confidence interval (CI), unless stated. Shapiro–Wilk W test was used for checking the normality of data distribution. Group comparisons at baseline were done by analysis of variance (ANOVA) for continuous variables and chi-square test for categorical variables. Between-variable association was evaluated using the Pearson correlation test. Multiple linear regression analysis was conducted to reveal the estimated effect of interventions with vitamin D on outcomes. All statistical analyses were performed with STATA Statistical Software release 16 (STATA, College Station, TX, USA). A *p* value of <0.05 was considered significant.

## Results

Data were collected from 75 participants (mean age: 50.7 ± 6.1 years) of whom 45 were women (60.0%). Three groups were similar in terms of distribution of age (*p* = 0.496) and gender (*p* = 0.513).

**Fig. 1:**
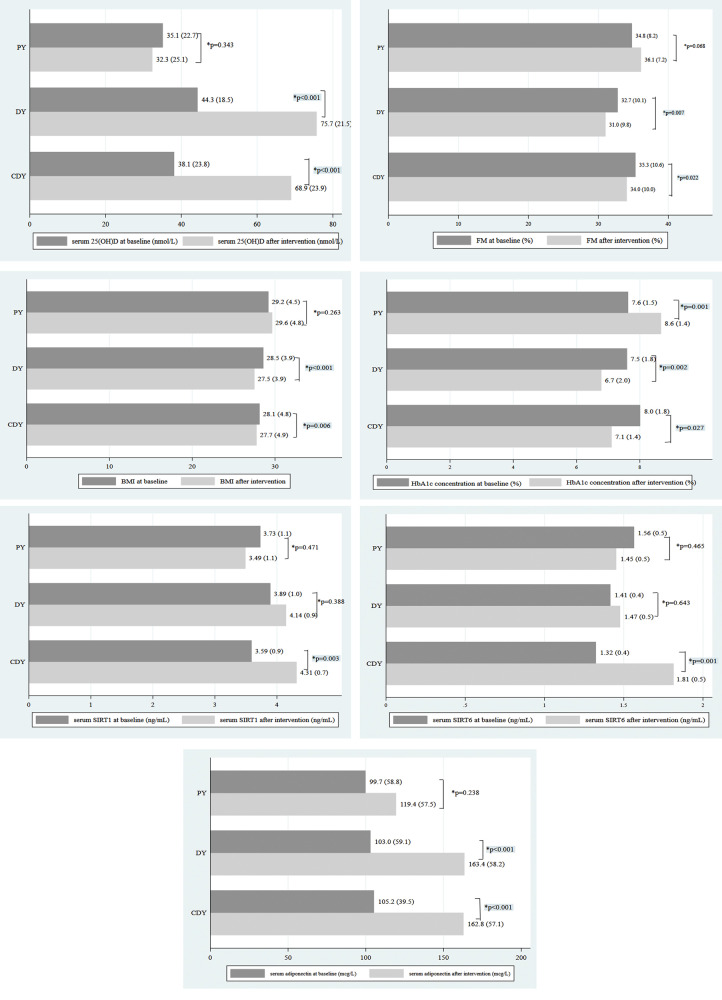
Comparison of mean variables in groups over time. *: Within-group comparison. FM Percent of total body fat mass, HbA1c hemoglobin A1c, 25(OH)D 25-hydroxycalciferol, SIRT sirtuin.

There were no significant within- or between-group differences in dietary intakes (Table 1). The distribution of the studied variables did not show any significant between-group difference at the baseline (Table 2). Serum 25(OH)D concentration significantly increased from baseline in both vitamin D-supplemented groups. A significant within-group increment in serum adiponectin concentrations was observed in both DY and CDY groups (+60.4 ± 8.6, +57.5 ± 6.4, respectively; *p* < 0.001 for both). However, the between-group difference was not statistically significant. When compared with baseline values, we found a significant decrease in BMI, FM, and HbA1c in both vitamin D-supplemented groups. Interestingly, the concentrations of SIRT1 and SIRT6 had a significant within-group increment compared to baseline, only in the CDY group (*p* = 0.003, *p* = 0.001 respectively), but not in DY or PY groups (Table 2). Figure 1 shows the comparison of mean variables in the studied groups over time.

**Table 1:** Comparison of mean daily intake of energy, macronutrients, calcium, and vitamin D^a^ between three groups.

Variables	PY	DY	CDY	*p* value^b^
*Energy (kcal)*				
Before	1684.3 ± 710.2	1659.9 ± 460.0	1584.3 ± 483.8	0.832
After	1631.4 ± 639.7	1662.5 ± 449.9	1507.6 ± 357.7	
*p* value^c^	0.642	0.978	0.223	
*Protein (g)*				
Before	69.7 ± 30.9	58.6 ± 19.4	58.3 ± 23.8	0.372
After	60.9 ± 20.6	63.6 ± 31.4	57.8 ± 21.1	
*p* value^c^	0.109	0.321	0.872	
*Carbohydrate (g)*				
Before	237.5 ± 106.4	253.7 ± 84.2	225.6 ± 79.8	0.653
After	211.1 ± 74.9	247.0 ± 91.6	211.0 ± 65.0	
*p* value^c^	0.117	0.719	0.201	
*Fat (g)*				
Before	53.0 ± 26.6	48.6 ± 14.1	53.3 ± 26.2	0.781
After	59.9 ± 42.9	55.9 ± 19.3	50.4 ± 18.1	
*p* value^c^	0.380	0.134	0.593	
*Fiber (g)*				
Before	18.9 ± 13.0	18.3 ± 7.1	16.4 ± 6.1	0.558
After	18.8 ± 8.1	19.8 ± 8.9	15.3 ± 6.4	
*p* value^c^	0.965	0.521	0.394	
*Calcium (mg)*				
Before	687.9 ± 280.4	714.4 ± 291.1	679.2 ± 262.3	0.807
After	687.6 ± 283.2	734.3 ± 311.7	670.8 ± 268.0	
*p* value^c^	0.996	0.779	0.875	
*Vitamin D (IU)*				
Before	34.6 ± 45.2	28.5 ± 25.4	22.1 ± 29.6	0.127
After	19.5 ± 8.0	21.7 ± 10.2	15.0 ± 5.6	
*p* value^c^	0.129	0.203	0.289	

*CDY* calcium+D -fortified yogurt drink, *DY* D-fortified yogurt drink, *PY* plain (unfortified) yogurt drink

^a^The added amounts of calcium and vitamin D to the yogurt drinks are not considered here

^b^Donated significance of between-group comparisons at baseline (one-way ANOVA)

^c^Donated significance of within-group comparisons (Paired sample *t* test)

**Table 2:** Comparison of mean variables in groups over time.

Variables	PY						DY	CDY	*p* value^a^
*BMI (kg/m*^*2*^*)*									
Before	29.2 ± 4.5						28.5 ± 3.9	28.1 ± 4.8	0.698
After	29.6 ± 4.8						27.5 ± 3.9	27.7 ± 4.9	
*p* value^b^	0.263						<0.001	0.006	
*FM (%)*									
Before	34.8 ± 8.2						32.7 ± 10.1	35.3 ± 10.6	0.624
After	36.1 ± 7.2						31.0 ± 9.8	34.0 ± 10.0	
*p* value^b^	0.068						0.007	0.0225	
*25(OH)D (nmol/L)*									
Before	35.1 ± 22.7						44.3 ± 18.5	38.1 ± 23.8	0.321
After	32.3 ± 25.1						75.7 ± 21.5	68.9 ± 23.9	
*p* value	0.343						<0.001	<0.001	
*HbAlc, %*									
Before	7.6 ± 1.5						7.5 ± 1.8	8.0 ± 1.8	0.656
After	8.6 ± 1.4						6.7 ± 2.0	7.1 ± 1.4	
*p* value^b^	0.001						0.002	0.027	
*Adiponectin (µg/L)*									
Before	99.7 ± 58.8						103.0 ± 59.1	105.2 ± 39.5	0.935
After	119.4 ± 57.5						163.4 ± 85.2	162.8 ± 57.1	
*p* value^b^	0.238						<0.001	<0.001	
*SIRT1 (ng/mL)*									
Before	3.73 ± 1.1						3.89 ± 1.0	3.59 ± 0.9	0.595
After	3.49 ± 1.1						4.14 ± 0.9	4.31 ± 0.7	
*p* value^b^	0.471						0.388	0.003	
*SIRT6 (ng/mL)*									
Before	1.56 ± 0.5						1.41 ± 0.4	1.32 ± 0.4	0.189
After	1.45 ± 0.5						1.47 ± 0.5	1.81 ± 0.5	
*p* value^b^	0.465						0.643	0.001	

*BMI* body mass index, *CDY* calcium+D-fortified yogurt drink, *DY* D-fortified yogurt drink, *FM* total body fat mass, *HbA1c* hemoglobin A1c, *25(OH)D* 25-hydroxy vitamin D, *PY* plain (unfortified) yogurt drink, *SIRT* sirtuin

^a^Between-group comparisons at baseline (one-way ANOVA)

^b^Within-group comparisons (Paired sample *t* test)

Table 3 demonstrates the results of Pearson correlation analysis between changes of the variables. SIRT1 was directly correlated with SIRT6 (*r* = 0.375, *p* < 0.001), 25(OH)D (*r* = 0.336, *p* = 0.003) and adiponectin (*r* = 0.300, *p* = 0.008) but inversely with HbA1c (*r* = –0.391, *p* = 0.001). Similarly, SIRT6 showed a direct correlation with 25(OH)D (*r* = 0.328, *p* = 0.004) but an inverse correlation with HbA1c (*r* = −0.252, *p* = 0.029). Serum 25(OH)D concentration was inversely correlated with HbA1c (*r* = −0.328, *p* < 0.001).

**Table 3:** The results of Pearson correlation analysis between changes of the studied variables.

Variable	SIRT1	SIRT6	HbA1c	25(OH)D	Adiponectin
SIRT6	0.375				
	<0.001				
HbA1c	−0.391	−0.252			
	0.001	0.029			
25(OH)D	0.336	0.328	−0.382		
	0.003	0.004	<0.001		
Adiponectin	0.300	0.116	−0.119	0.250	
	0.008	0.321	0.261	0.017	
FM	−0.100	−0.102	0.176	−0.249	−0.142
	0.390	0.381	0.096	0.018	0.180

*FM* percent of total body fat mass, *HbA1c* hemoglobin A1c, *25(OH)D* 25-hydroxycalciferol, *SIRT* sirtuin

Table 4 shows the results of the regression models adjusted for baseline values indicating that daily intake of D-fortified yogurt drink either with (*B* = 0.93, *p* < 0.001) or without added calcium (*B* = 0.52, *p* = 0.002) could increase SIRT1 concentrations compared with PY. Moreover, being in each of the vitamin D-supplemented groups, compared with PY, had a significant effect on serum concentrations of 25(OH)D and adiponectin, as well as on BMI and FM after 12 weeks intervention period. Likewise, being in the CDY group, as compared to the PY group, had a significant effect on serum concentrations of SIRT6. The pairwise comparisons after adjustment for multiple comparison (Tukey’s) indicated that among subjects in both D-fortified groups, being in the CDY group was more favorable predictor of improvement in SIRT6 concentrations as compared with the DY (*p* = 0.007) and PY (*p* < 0.001) (Table 3).

**Table 4:** Multiple regression results for variables after intervention.

	Variables	*B*	St. error	*p* value	95% CI	*r*^2^
BMI	PY	–	–	–	–	0.93
	DY [1, 3]	−1.5	0.3	<0.001	−2.2 to −0.8	
	CDY [1, 2]	−0.8	0.3	0.02	−1.5 to −0.1	
FM	PY	–	–	–	–	0.91
	DY [1]	−3.2	0.7	<0.001	−4.8 to −1.6	
	CDY [1]	−2.4	0.7	0.002	−4.0 to −0.9	
25(OH)D	PY	–	–	–	–	0.68
	DY [1]	36.5	4.9	<0.001	26.6–46.3	
	CDY [1]	34.3	4.8	<0.001	24.6–44.1	
HbA1c	PY	–	–	–	–	0.48
	DY [1]	−1.8	0.3	<0.001	−2.6 to −1.1	
	CDY [1]	−1.7	0.3	<0.001	−2.5 to −1.0	
Adiponectin	PY	−	−	−	−	0.7
	DY [1]	40.6	10.9	<0.001	18.8–62.4	
	CDY [1]	37.6	10.9	0.001	15.8–59.4	
SIRT1	PY	–	–	–	–	0.71
	DY [1, 3]	0.52	0.16	0.002	0.2–0.84	
	CDY [1, 2]	0.93	0.16	<0.001	0.61–1.25	
SIRT6	PY	–	–	–	–	0.37
	DY [3]	0.12	0.12	0.344	−0.13 to 0.37	
	CDY [1, 2]	0.51	0.12	<0.001	0.26–0.77	

Model 1: adjusted for value of variables before interventions

*BMI* body mass index, *CDY* calcium+D-fortified yogurt drink, *DY* D-fortified yogurt drink, *FM* total body fat mass, *HbA1c* hemoglobin A1c, *25(OH)D* 25-hydroxy vitamin D, *PY* plain (unfortified) yogurt drink, *SIRT* sirtuin

Table 5 displays the results of the regression models incorporating changes of 25(OH)D, serum SIRT1 and SIRT6 individually with changes of HbA1c as outcome and also the regression models incorporating these variables with changes of serum adiponectin and changes of FM as outcomes. The models showed that changes of serum 25(OH)D was a significant predictor of changes of serum adiponectin, HbA1c and FM. However, the association between changes of circulating 25(OH)D and adiponectin disappeared when it was adjusted for changes of serum SIRT1. In contrast, the association between changes of circulating 25(OH)D and HbA1c remained significant even after adjustment for SIRT1. There were no associations between changes in serum SIRT6 and changes in HbA1c and changes in serum adiponectin.

**Table 5:** Multiple regression results for variables after intervention.

Dependent variables	Predictors	Models	B	St. error	p value	95% CI	adjusted *r*^2^
Changes of FM	Changes of 25(OH)D	Unadjusted	−0.03	0.01	0.018	−0.05 to −0.005	
		Model^a^	−0.03	0.51	0.008	−0.06 to −0.01	0.10
		Model^b^	−0.03	0.01	0.008	−0.06 to −0.01	0.10
	Changes of SIRT1	Unadjusted	−0.44	0.51	0.391	−1.4 to 0.57	
		Model^a^	0.03	0.51	0.943	−0.99 to 1.07	0.10
	Changes of SIRT6	Unadjusted	−0.62	0.70	0.381	−2.02 to 0.785	
		Model^b^	0.01	0.71	0.979	−1.4 to 1.44	0.10
Changes of HbA1c	Changes of 25(OH)D	Unadjusted	−0.02	0.006	<0.001	−0.03 to −0.01	
		Model^a^	−0.01	0.006	0.061	−0.02 to 0.001	0.23
		Model^b^	−0.02	0.007	0.011	−0.03 to 0.004	0.17
	Changes of SIRT1	Unadjusted	−0.94	0.26	0.001	−1.4 to −0.42	
		Model^a^	−0.05	0.22	0.795	−0.51 to 0.39	0.05
	Changes of SIRT6	Unadjusted	−0.84	0.37	0.029	−1.60 to −0.08	
		Model^b^	−0.49	0.38	0.204	−1.25 to 0.273	0.17
Changes of adiponectin	Changes of 25(OH)D	Unadjusted	0.38	0.15	0.017	0.06 to 0.69	
		Model^a^	0.114	0.19	0.549	−0.26 to 0.49	0.12
		Model^b^	0.23	0.19	0.250	−0.16 to 0.61	0.06
	Changes of SIRT1	Unadjusted	17.3	6.4	0.009	4.47–30.1	
		Model^a^	15.6	6.8	0.026	8.91–29.2	0.12
	Changes of SIRT6	Unadjusted	9.2	9.2	0.321	−9.2 to 27.7	
		Model^b^	5.1	9.7	0.604	−14.3 to 24.5	0.06
Changes of Sirtuin 1	Changes of 25(OH)D	Unadjusted	0.008	0.002	0.003	0.003–0.014	0.11
		Model^c^	0.006	0.003	0.047	0.0001– 0.012	0.17
Changes of Sirtuin 6	Changes of 25(OH)D	Unadjusted	0.006	0.002	0.004	0.002–0.01	0.10
		Model^c^	0.005	0.002	0.02	0.001–0.01	0.10

*FM* total body fat mass, *HbA1c* hemoglobin A1c, *25(OH)D* 25-hydroxy vitamin D, *SIRT* sirtuin

^a^Variables in model 1: changes of serum 25(OH)D, changes of serum SIRT1

^b^Variables in model 2: changes of serum 25(OH)D, changes of serum SIRT6

^c^Variables in model 3: changes of serum 25(OH)D, changes of blood Hb A1c, changes of fat mass

In univariate regression, changes of circulating 25(OH)D was a significant determinant of FM changes (*B* = −0.03, 95%CI = −0.05 to −0.005, *p* = 0.018). Likewise, in multiple regression analysis, serum 25(OH)D remained significant predictor of FM even after adjustment for changes of serum concentrations of SIRT1 (*B* = −0.03, 95%CI = −0.06 to −0.01, *p =* 0.008) and SIRT6 (*B* = −0.03, 95%CI = −0.06 to −0.01, *p* = 0.008).

## Discussion

Our findings showed that improvement of vitamin D status via daily intake of Ca+D-yogurt drink resulted in a significant increase in serum concentrations of sirtuins 1 and 6. These findings are in accord with several experimental studies. For example, in rats fed on low vitamin D diet, secretion of SIRT1 was decreased [43] whereas in mice fed on high-fat diet, vitamin D_3_ supplementation resulted in up-regulation of SIRT1 [44]. Current evidence shows that the association of vitamin D and sirtuins may be through both direct and indirect pathways. Direct association of vitamin D with SIRT1, through vitamin D receptor (VDR), has been shown in experimental models [44–47]. On the other hand, vitamin D-induced up-regulation of SIRT1 together with pAMPK and GLUT-4 in adipose tissue suggests a role for these insulin-independent signaling molecules in glycemic control through vitamin D [44].

Sirtuin 6 contributes to glucose homeostasis by enhancing insulin secretion and inhibiting gluconeogenesis as well as lipogenesis [37]. In macrophages, SIRT6 suppresses obesity-induced inflammation and insulin resistance [32]. In fat-specific SIRT6 knockout mice fed on a high-fat diet, there was an augmented tendency to obesity, inflammation, and insulin resistance. An under-expression of SIRT6 and related reduced adipose triacylglycerol lipase activity was observed in obese subjects [48]. Nevertheless, various studies have documented that both inhibition and enhancing SIRT6 may improve glucose tolerance in T2D. In the murine model of T2D, inhibition of SIRT6 for ten days resulted in over-expression of muscular GLUT-1 and GLUT-4, enhanced glycolysis, decreased serum insulin as well as blood lipid concentrations and improved oral glucose tolerance [49]. On the other hand, experimental studies provided strong evidence for SIRT6 in pancreatic β-cells function [50, 51]. Notwithstanding, our findings provide clinical evidence for vitamin D-induced increased SIRT6, which was accompanied by a formerly reported significant improvement of glycemic status in T2D subjects [15].

We found consuming yogurt drink fortified with both vitamin D and calcium is more favorable to increase SIRT6. It has been shown that mitochondrial matrix calcium has a regulatory effect on sirtuin expression [52]. The effect of calcium intake on different aspects of diabetes including body weight and insulin resistance has been vastly studied [53–55] but still is controversial [56]. It is noteworthy that the mean calcium intake in our participants was about 700 mg/d, considerably less than the recommended intake for this age group. It is therefore likely that supplementing calcium intake in the CDY group might, at least in part, contribute to its SIRT6 enhancing effect.

Disappearance of the association of changes of serum 25(OH)D and adiponectin concentrations following adjustment for changes of serum SIRT1 indicates a SIRT1-mediated effect of vitamin D on adiponectin secretion. Thus, vitamin D up-regulates SIRT1, as demonstrated in both animal model [44] and randomized clinical trial [25] and then SIRT1 in turn regulates adiponectin secretion. This finding is in accord with the report of regulation of adiponectin secretion by SIRT1 and endoplasmic reticulum oxidoreductase Ero1-Lα [57]. It is also documented that SIRT1 can potentiate 1,25-dihydroxycalciferol, the active form of vitamin D, via enzymatic deacetylation of VDR [58].

In the current study, there was a significant decrease in BMI in both vitamin D-supplemented groups despite no significant change in energy intake during 12 weeks intervention period. Though the enhancing effect of dairy calcium intake on weight loss in subjects with diabetes has already been reported [59], we found no significant change in the PY group. Along the same line, a prospective study in Australia demonstrated an association of higher circulating 25(OH)D concentrations, but not dietary calcium intake, with a lower risk of diabetes in adults [60].

In this study, reduction of BMI in both vitamin D supplemented groups was independent of changes of serum SIRT1 and SIRT6 concentrations. In oppose to this finding, it has been shown that vitamin D may have a fat-storing inhibitory effect on adipocytes which is mediated by NAD and SIRT1 [61]. It is, therefore, likely that the effect of vitamin D on body weight may be mediated through both SIRT1-dependent and SIRT1-independent pathways. In a study, adipocyte and muscle cell culture media were treated by adding sera obtained from the overweight/obese subjects fed a low or high-dairy diet for four weeks. The results demonstrated activation of SIRT1 and SIRT1-independent pathways in media treated with high-dairy dieters’ sera resulting in enhanced mitochondrial biogenesis [62]. The regulatory action of SIRT1 on energy metabolism has been reported earlier [63].

The limitations of the present study must be acknowledged. Firstly, the short-term effects observed in this study do not necessarily reflect any possible long-term effects. Secondly, the other sirtuins with a possible effect on pancreatic β-cell function, notably SIRT3 [64, 65], were not examined, either.

## Conclusions

On the whole, daily consumption of vitamin D-fortified yogurt drink for 12 weeks resulted in an increase in circulating concentrations of SIRT1 and SIRT6 in T2D subjects and D+Ca-fortified, as compared with only D-fortified, yogurt drink was more in favor of SIRT6 increment. It is likely that the improving effect of vitamin D on adiponectin is SIRT1-dependent whereas its effect on HbA1c is SIRT1-independent. These findings shed some light on the mechanism of action of vitamin D on different aspects of diabetes including body weight and glycemic status.

## Supplementary information

CONSORT checklist

## Data Availability

All data generated are included in this published article.
